# A Generalized Helfrich Free Energy Framework for Multicomponent Fluid Membranes

**DOI:** 10.3390/membranes15060182

**Published:** 2025-06-17

**Authors:** Hao Wu, Zhong-Can Ou-Yang

**Affiliations:** 1 Wenzhou Institute, University of Chinese Academy of Sciences, Wenzhou 325001, China; 2 Institute of Theoretical Physics, Chinese Academy of Sciences, Beijing 100190, China; oy@itp.ac.cn

**Keywords:** multicomponent membranes, curvature-component coupling, generalized Helfrich free energy, Euler-Lagrange equations

## Abstract

Cell membranes contain a variety of biomolecules, especially various kinds of lipids and proteins, which constantly change with fluidity and environmental stimuli. Though Helfrich curvature elastic energy has successfully explained many phenomena for single-component membranes, a new theoretical framework for multicomponent membranes is still a challenge. In this work, we propose a generalized Helfrich free-energy functional describe equilibrium shapes and phase behaviors related to membrane heterogeneity with via curvature-component coupling in a unified framework. For multicomponent membranes, a new but important Laplace–Beltrami operator is derived from the variational calculation on the integral of Gaussian curvature and applied to explain the spontaneous nanotube formation of an asymmetric glycolipid vesicle. Therefore, our general mathematical framework shows a predictive capabilities beyond the existing multicomponent membrane models. The set of new curvature-component coupling EL equations have been derived for global vesicle shapes associated with the composition redistribution of multicomponent membranes for the first time and specified into several typical geometric shape equations. The equilibrium radii of isotonic vesicles for both spherical and cylindrical geometries are calculated. The analytical solution for isotonic vesicles reveals that membrane stability requires distinct elastic moduli among components ($k_{A}\neq k_{B}$, ${\bar{k}}_{A}\neq {\bar{k}}_{B}$), which is consistent with experimental observations of coexisting lipid domains. Furthermore, we elucidate the biophysical implications of the derived shape equations, linking them to experimentally observed membrane remodeling processes. Our new free-energy framework provides a baseline for more detailed microscopic membrane models.

## 1. Introduction

Living cells are encapsulated by a lipid bilayer that separates the interior contents of the cell from the exterior environment. Phospholipids are the main molecules in the membrane bilayer. They are polar; thus, the apolar tails orient away from the polar environment. Under certain concentrations, the lipid bilayer in the water will bend automatically to form a giant vesicle, which is usually used as a basic prototype model of the cell [1,2].

Since Simons [3] proposed the new concept—functional rafts floating on cell membranes four decades ago—the heterogeneity of cell membranes has attracted interdisciplinary attention. Extensive biochemical experiments and theoretical studies have been conducted to explore this phenomenon. It has been realized that the lipid–lipid immiscibility gives rise to lateral heterogeneity in the plane of plasma membranes—the subsets of which are termed lipid rafts. Originally defined biochemically as detergent-resistant membrane (DRM) fractions [4] or detergent-insoluble glycolipid-enriched membrane (DIG) domains [5,6,7], lipid rafts are proposed to be highly dynamic submicroscopic assemblies that float freely within the liquid-disordered bilayer in cell membranes and can coalesce upon clustering of their components. DRMs and DIGs have a greater bending rigidity [8], as well as a much larger area incompressibility [9] than the surrounding unsaturated lipid matrix [10]. Two physiologically important processes that occur in biological membranes are the partition of water-soluble proteins in the membrane and the sequestration of specific transmembrane proteins into membrane microdomains or ‘rafts’ [11]. They both strongly depend on the structural and elastic properties of the membrane bilayer. The raft hypothesis [12] proposes that certain naturally occurring lipids aggregate in the membrane plane driven solely by distinctive intermolecular interactions, including van der Waals interactions between long, nearly fully saturated chains of sphingomyelin and glycosphingolipids, as well as hydrogen bonding between adjacent glycosyl moieties of glycosphingolipids [13]. The potential of glycosphingolipids to form domains was recognized earlier [14]. Furthermore, the saturated nature of the lipids and glycolipids in the raft acts to promote their interaction with cholesterol [15].

In in vitro experiments [16,17], multicomponent giant unilamellar vesicles (GUVs) have been widely applied to mimic realistic cell membranes and their dynamics, such as budding [18] and fission [19]. It has been confirmed by experiments and simulations that cell membranes are indeed laterally heterogeneous on scales that appear to range from hundreds of nanometers to a few micrometers [6,7,20]. Heterogeneities on this scale are commonly referred to as microdomains. It appears that membrane lipid microdomains containing hundreds to tens of thousands of phospholipid molecules are more likely to form as a result of protein–lipid interactions than as a result of lipid–lipid immiscibility or phase separation. Some experiments detected membrane domains arising from protein–protein interactions [21]; therefore, the domains are considered as two-dimensional solid plate-like colloidal islands floating on the fluid membrane sea [22], in which rich interactions emerge. In nature, its counterpart has been found, wherein caveolae are small flask-shaped invaginations of cell membranes, 70–100 nm in diameter, which are coated with caveolin—a protein that binds cholesterol [23]. Caveolae are enriched in cholesterol and glycosphingolipids as well as in glycosylphosphatidyl inositol (GPI)-anchored proteins [24].

Since the original Helfrich curvature elastic free energy was proposed for single-component lipid membranes in 1973 [25], three main continuum derivative models have been developed to simulate the shape transformation in red blood cells (RBCs) and other cells. The first type of model was developed to describe various shapes of cells and vesicles: one is called the area difference elasticity (ADE) model [1], which is based on the bilayer couple hypothesis. In this model, the area difference between the outer and inner lipid bilayer is taken as a dominant parameter for shape transformations from the stomatocyte toward the discocyte and finally to the echinocyte. The second model is based on the bilayer skeleton couple assumption. In this model, the membrane skeleton is believed to play an active role in controlling shape changes, and the coupled influences of the bilayer and the cytoskeletal network are taken into account at the same time. By using this model, echinocytes with various numbers and lengths of spicula can be simulated [26]. Recently, a third type of model with locally variable elastic moduli has been proposed by taking account of cell membrane heteorgeneities with the reaction from the cytoskeleton underneath [27]. The asymmetric pear shape, stomach shape, and kidney shape have been observed in this model by simulation. In addition to the continuum model, the discrete particle-based simulation models, such as dissipative particle dynamics (DPD) [28] and coarse-grained Langevin dynamics (CLD) [6,7], are widely used to simulate the multicomponent membranes as well.

Although the above models have made considerable exciting progress in different directions, a comprehensive continuous model, which can quantitatively describe the dynamics of shape change and composition redistribution at equilibrium, is still worth developing. For this purpose, a continuous model combined with Ginzburg–Landau free energy was first constructed by Taniguchi [29], which was later followed by others in the same way [30,31,32,33,34]. Limited to the assumption of homogeneous membranes and the loss of two geometric constraints (area and volume), the model with no unique solution was not comparable to the experiments (see Section 2.2), but it opened a path to a generalized Helfrich framework. In the spirit of the above model, a new continuous model for the equilibrium shapes and curvature-coupled composition redistribution of multicomponent membranes is presented in this article by thoroughly considering membrane heterogeneity, membrane composition, and the geometric constraints for the total area and total volume. In Section 2, we discuss the new generalized Helfrich functional and the importance of the geometric constraints. In Section 3, we calculate the geometrically constrained Euler–Lagrange equations (equilibrium shape equations) associated with the composition redistribution to describe membrane deformation with the corresponding phase separation on the membrane surface. In Section 4, several typical geometric surfaces with the symmetry are considered to obtain the analytical forms for the shape equations. In Section 5, we conclude the advantage of the current model and discuss the future development and some applications.

## 2. Generalized Helfrich Free Energy for Multicomponent Heterogeneous Vesicles

### 2.1. Formulation of Generalized Helfrich Free Energy

**Figure 1:**
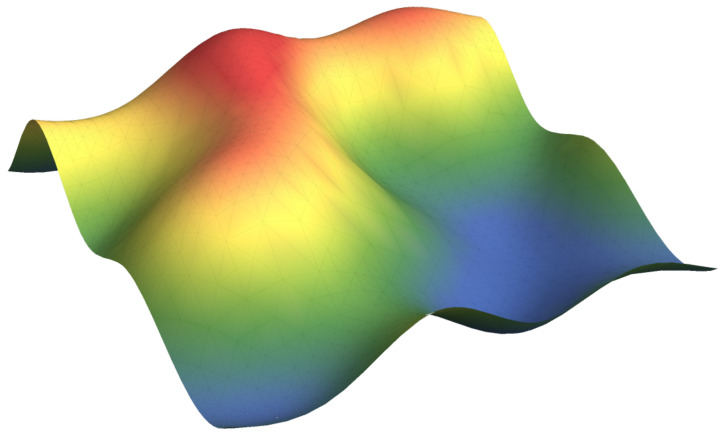
A schematic figure for multicomponent membranes is plotted, and the color map represents the variation of lipid components.

Based on the classic Helfrich free energy [25] with constant bending rigidity for homogeneous membranes and the framework later developed by Ou-Yang [35] that calculated the equilibrium shape equation with the area and volume constraints by introducing membrane tension and cross-membrane pressure drop, in this paper, we further consider all these elastic moduli, such as bending rigidity, Gaussian bending rigidity, and membrane tension, variable with lipid components on the membrane surface as shown in Figure 1. The general energy functional for multicomponent membranes includes three contributions:

(1) $$F=F_{p}+F_{s}+F_{v},$$

The first functional is related to the variation of membrane components on the surface

(2) $$F_{p}=\frac{\mu }{2}\int {\left(\nabla_{s}\phi \right)}^{2}dA,$$

where μ is usually a quadratic function of the correlation length ξ, which determines the interface width. The second one is similar to the classic Helfrich free energy and characterizes the elasticity of multicomponent membranes as

(3) $$F_{s}=\int \left[\frac{k(\phi )}{2}{\left(2H\right)}^{2}+\bar{k}\left(\phi \right)K+\sigma \left(\phi \right)+V\left(\phi \right)\right]dA,$$

with the free-energy integral kernel

(4) $$G\left(H,K,\phi \right)=\frac{k(\phi )}{2}{\left(2H\right)}^{2}+\bar{k}\left(\phi \right)K+\sigma \left(\phi \right)+V\left(\phi \right),$$

where k(ϕ) and $\bar{k}\left(\phi \right)$ are the variable bending modulus and the variable Gaussian modulus, and σ(ϕ) is the component-dependent membrane tension to conserve the local membrane area. The last integration is a symmetric double-well potential defined as

(5) $$V\left(\phi \right)=\frac{\lambda }{2}\phi^{2}{\left(1-\phi \right)}^{2},$$

whose details are not that important [36], and λ is the coupling coefficient between different components. Such a $\phi^{4}$ potential V(ϕ) and the quadratic form of $\nabla_{s}\phi$ in Equation (2) considered together are usually considered as the classic Ginzburg–Landau free-energy framework to quantitatively describe the phase separation and the interface between different phases. ϕ is an order parameter and represents either the relative molar fraction of two phase components A and B of the lipid membrane: $\phi =\left(\phi_{A}-\phi_{B}\right)/\left(\phi_{A}+\phi_{B}\right)=\phi_{A}-\phi_{B}$, with $\phi_{A}+\phi_{B}=1$, or the molar fraction or concentration of a diffusing membrane component $\phi =\phi_{X}$ (X is A, B,…), such as one type of lipids—glycolipids [6,7,37]—or transmembrane proteins [32], on the membrane surface, or both of them. For simplicity, let us consider $\phi =\phi_{A}$, where $\phi_{A}$ represents the molar fraction of lipid A; then, the double-well potential can be considered as the mixing entropy of a two-component membrane system as the term in Flory–Huggins theory for two-component polymers [38] as follows:

(6) $$V\left(\phi_{A}\right)=\frac{\lambda }{2}\phi_{A}^{2}\phi_{B}^{2}=\frac{\lambda }{2}\phi_{A}^{2}{\left(1-\phi_{A}\right)}^{2},$$

As opposed to the Canham–Helfrich approach recently reviewed in the previous reference [2], our model explicitly takes into account a diffusive interface. This is different from the previous work dealing with two-component membranes in a strong segregation limit, where the components are well separated with a well-defined boundary between two domains [39]. Our model can appropriately describe a transition process from weak segregation to strong segregation; the interfacial line, therefore, can emerge naturally in our model as the interface width decreases. The interface width *d* can be calculated as $d=4\sqrt{\mu /\lambda }$ [40], which indicates that the interface width between two phases increases with μ but decreases with λ. λ directly controls the depth of the potential wells. As λ increases, the energy barrier $V_{\max}$ rises, leading to stronger phase separation (sharper domain interfaces). λ is directly linked to the interfacial line tension between phases. Using classical phase field theory (Cahn–Hilliard model), the interfacial tension can be expressed as $\sqrt{\mu \lambda }/6$, where μ is the gradient energy coefficient (Equation (2)). This indicates that increasing λ not only enhances phase separation but also elevates the interfacial line energy, suppressing interface fluctuations. λ generally decreases with temperature due to enhanced mixing entropy (analogous to the Flory–Huggins parameter χ). For isothermal systems, $\lambda \left(T\right)=\lambda_{0}\left(1-T/T_{c}\right)$, where $T_{c}$ is the critical phase separation temperature. In cell membranes, λ regulation has functional implications. High λ (strong phase separation) stabilizes lipid rafts (cholesterol/sphingolipid-rich domains), which is critical for signaling and protein sorting. Diseases like atherosclerosis correlate with aberrant λ values due to oxidized lipids, which disrupt phase separation [41].

The Landau $\phi^{4}$ theory for phase separation can be extended to three or more component systems by redefining the order parameters, free-energy functionals, and dynamical equations to account for multicomponent interactions. In general, for an *N*-component system, N−1 independent order parameters are required. Here, we briefly introduce how to extend our framework to ternary fluid membranes. Let $\phi_{1}$ be the oncentration difference between components A and B, and $\phi_{2}$ is the concentration difference between component C and the (A + B) mixture. The free-energy density for a ternary system includes cross-coupling terms:

(7) $$f\left(\phi_{1},\phi_{2}\right)=\frac{\alpha }{2}\left(\phi_{1}^{2}+\phi_{2}^{2}\right)+\frac{\beta }{4}\left(\phi_{1}^{4}+\phi_{2}^{4}\right)+\gamma \phi_{1}^{2}\phi_{2}^{2}+\frac{\kappa }{2}\left[{\left(\nabla \phi_{1}\right)}^{2}+{\left(\nabla \phi_{2}\right)}^{2}\right]$$

where α<0 drives phase separation, β>0 stabilizes double-well potential, γ controls phase separation patterns, and κ is the gradient energy coefficient. The total concentration conservation is $\sum_{i=1}^{N}\phi_{i}=1$. In the ternary system, $\gamma_{\mathrm{crit}}=\beta /2$, $\gamma <\gamma_{\mathrm{crit}}$ indicates two-phase coexistence (e.g., A-B vs. C), while $\gamma >\gamma_{\mathrm{crit}}$ indicates three-phase coexistence (e.g., A vs. B vs. C).

If a closed multicomponent vesicle is considered, we still need the third functional for the global volume constraint

(8) $$F_{v}=\int PdV=P\int dV$$

where *P* is the cross-membrane pressure difference, and it is a homogeneous elastic property, because it always can relax to the equilibrium state instantaneously [27].

### 2.2. Importances of Two Geometric Constraints

As far as we know, the relation between the constraints and the minimization of Helfrich free energy have not been fully discussed. To answer this problem, we use the symmetric version (zero spontaneous curvature [1]) of Helfrich free energy $H_{s}=\int 2\kappa H^{2}dA$, as has been used in many references [1,26,42,43,44] without the loss of generality. In terms of Noether’s theorem, the symmetric Helfrich free energy, quadratic in the extrinsic curvature *H*, conserves of the stress tensor: The normal projection is identified as the shape equation describing equilibrium configurations; the tangential projections are consistency conditions on the stresses which capture the fluid character of such membranes, and the corresponding torque tensor is identified. The conservation laws are cast in terms of the forces and torques on closed curves embedded in the membrane surface. As an application, the first integral of the shape equation for axially symmetric configurations can be derived from it. This version is also a scale-invariant energy. For the case of no constraint, the problem in the absence of external forces becomes exactly a standard mathematical problem of Willmore surfaces, whose well-known conclusions have shown a sphere as the stable shape for genus g=0 (spherical topology) and a Clifford torus for genus g=1 (toroidal topology). (We see that the stable shape without any constraint is only dependent of the genus.)That is why Taniguchi’s simulation produces only a spherical vesicle [29]. When we add one constraint, for example, the area, the scaling argument indicates that a single constraint has no effect on the shapes in equilibrium (for a mathematically differentiable surface, the thermal fluctuation is not considered), just like the case of no constraint. To see this, let us add an area conservation term $\Lambda A\left(r\right)$; then, $H\left(r\right)=H_{s}\left(r\right)+\Lambda A\left(r\right)$. (If the area constraint is written in the form of $\Lambda \left[A\left(r\right)-A_{0}\right]$, then under the scaling procedure, we can obtain $\Lambda \left[{\left(1+\lambda \right)}^{2}A\left(r\right)-A_{0}\right]$ with the scaling factor λ inside the square brackets, which, however, does not change the conclusion.) Under the rescaling transformation $r\to \left(1+\lambda \right)r$, where λ is constant, then $H\left[\left(1+\lambda \right)r\right]=H_{s}\left(r\right)+\Lambda {\left(1+\lambda \right)}^{2}A\left(r\right)$. And dH/dλ=0 when λ=0 in equilibrium implies that Λ=0. We can likewise analyze the case of the only volume constraint to obtian P=0. (Note that if ones consider the effect of the spontaneous curvature $c_{0}$, the Helfrich free energy should be rewritten as an asymmetric form (non-zero spontaneous curvature [1]) $H_{a}=\int \left[\kappa {\left(2H-c_{0}\right)}^{2}/2\right]dA$, in which the scale invariance is broken, and the above argument that Λ=0 with the area constraint alone is not valid any more. A remedy is to suppose that $c_{0}\to c_{0}/\left(1+\lambda \right)$ under the rescaling transformation. Then, the asymmetric Helfrich free energy is still the scale invariant.) In conclusion, if Helfrich free energy has only the volume constraint or the area constraint, the landscape of free energy has the only minimum with the shape of sphere in three dimensions (circle in two dimensions), in which case the minimum is both local and global. Based on the above condition, we add a new constraint on the enclosed volume $PV\left(r\right)$, which is competing with the area constraint; as a result, we have 2ΛA+3PV=0 in equilibrium. We find that this time the constraints matter. The coexistence of area and volume constraints introduces multiple local minima in the Helfrich free-energy landscape. Each equilibrium shape corresponds to a specific pair of Lagrange multipliers that enforce these constraints. Therefore, the geometric constraints are significant to obtain the realistic morphologies of vesicles and cells to compare with the experiments.

## 3. Calculation of Euler–Lagrange Equations

### 3.1. Variation for the Material Concentration on the Membrane: δϕ=Φ,δr=0

(9) $$\begin{array}{cc} \delta F & =\delta \int G\left(H,K,\phi \right)dA+\frac{\mu }{2}\delta \int {\left(\nabla_{s}\phi \right)}^{2}dA\\ \; & =\int \left(G_{\phi }-\mu \nabla_{s}^{2}\phi \right)\Phi dA\\ \; & =0 \end{array}$$

where in the second last step we applied the divergence theorem (also called Gauss’s theorem): There is no boundary on a closed vesicle, so the line integral is zero. Therefore, we obtain the first Euler–Lagrange equation as

(10) $$G_{\phi }-\mu \nabla_{s}^{2}\phi =G_{\phi }-\mu \Delta_{s}\phi =0$$

where $\nabla_{s}$ is the surface gradient operator, and $\Delta_{s}$ is the surface Laplacian operator or the Laplace–Beltrami operator.

### 3.2. Variation for the Deformation on the Membrane: $\delta \phi =0,\delta r=\phi^{i}e_{i}+\psi n$

Following the general variational principle for Helfrich shape equations [27,35,45,46,47], we calculate the calculus of variations in two virtual displacement directions: the normal one and the tangential one. For the derivation, we introduce the following symbols and definitions. The unit normal vector n points outward. The displacement of the surface is $dr=du_{1}e_{1}+du_{2}e_{2}+du_{3}n$. *g* is the surface metric tensor, and *L* is the extrinsic curvature tensor. $\psi_{,j}\equiv \partial_{j}\psi$, so $\nabla_{i}\psi_{,j}=\psi_{,ij}-\Gamma_{ij}^{k}\psi_{,k}=\partial_{i}\psi_{,j}-\Gamma_{ij}^{k}\psi_{,k}$, where $\Gamma_{ij}^{k}$ is the Christoffel symbol of the second kind, which is symmetric. $H=(\kappa_{1}+\kappa_{2})/2$ is the mean curvature, and $K=\kappa_{1}\kappa_{2}$ is the Gaussian curvature, where $\kappa_{1}$ and $\kappa_{2}$ are the principal curvatures. Two surface Laplacians (Laplace–Beltrami operators) are $\Delta_{s}\equiv \nabla_{s}^{2}\equiv {\left(\sqrt{g}\right)}^{-1}\partial_{i}\left(\sqrt{g}g^{ij}\partial_{j}\right)$ and ${\bar{\Delta }}_{s}\;={\bar{\nabla }}_{s}^{2}\equiv {\left(\sqrt{g}\right)}^{-1}\partial_{i}\left(\sqrt{g}KL^{ij}\partial_{j}\right)$, and i,j=1,2 throughout.

The second Laplace–Beltrami operator ${\bar{\Delta }}_{s}$ is not as well known as the first one $\Delta_{s}$, but it has a significant connection to the local topological changes of surfaces and can be used to explain a recent experiment on spontaneous nanotube formation [48]. The formation of nanotubes requests an abrupt change in the derivative of Gaussian bending rigidity. To avoid the ${\bar{\Delta }}_{s}\bar{k}\left(\phi \right)$ being divergent, mathematically, we need the Gaussian curvature K→0. This implies that surfaces with zero Gaussian curvature are the favorite local topological structures for local formation of tubular structures. In differential geometry, there are two important surfaces with zero Gaussian curvature: cylindrical surfaces and conical surfaces. For example, our mathematical framework allows echinocytes to form cylindrical or conic spicules. Our mathematical framework has the potential to explain the formation of nanotubes from vesicles as well as echinocytes with spiculated surface. This demonstrates new predictive capabilities of our model beyond the existing multicomponent membrane models.

Using covariant geometry through a series of increasingly tedious calculations, we can obtain the first variational equalities in both the normal and tangential directions as follows [27]:

$$\left\{\begin{array}{c} \delta r=\phi^{i}e_{i}+\psi n\\ \delta e_{i}=\left(\nabla_{i}\phi^{k}-\psi L_{i}^{k}\right)e_{k}+\left(\phi^{k}L_{ki}+\psi_{,i}\right)n\\ \delta n=-\left(\phi^{k}L_{k}^{i}+\nabla^{i}\psi \right)e_{i}\\ \delta g_{ij}=\nabla_{i}\phi_{j}+\nabla_{j}\phi_{i}-2\psi L_{ij}\\ \delta g^{ij}=-\nabla^{i}\phi^{j}-\nabla^{j}\phi^{i}+2\psi L^{ij}\\ \delta L_{ij}=\left(\nabla_{i}\nabla_{j}-2HL_{ij}+Kg_{ij}\right)\psi +L_{ik}\nabla_{j}\phi^{k}+L_{kj}\nabla_{i}\phi^{k}+\phi^{k}\nabla_{k}L_{ij}\\ \delta g=g\left(2\nabla_{i}\phi^{i}-4H\psi \right)\\ \delta \sqrt{g}=\sqrt{g}\left(\nabla_{i}\phi^{i}-2H\psi \right)\\ \delta H=\left(2H^{2}-K\right)\psi +\frac{1}{2}\Delta_{s}\psi +\phi^{i}\nabla_{i}H\\ \delta K={\bar{\Delta }}_{s}\psi +2HK\psi +\phi^{i}\nabla_{i}K \end{array}\right.$$

Applying the above expression, we can calculate the variations of Equations (2) and (3):

(11) $$\delta F=\delta F_{p}+\delta F_{s}$$

After a straightforward calculation for each term, we can integrate and reduce them as follows:

(12) $$\begin{array}{cc} \delta F & =\delta F_{s}+\delta F_{p}\\ \; & =\int \left[\frac{\Delta_{s}G_{H}}{2}+{\bar{\Delta }}_{s}G_{K}+\left(2H^{2}-K\right)G_{H}+2KHG_{K}-2HG\right]\psi dA\\ \; & -\int G_{\phi }\left(\phi^{i}\nabla_{i}\phi \right)dA+\mu \int \left(\nabla^{2}\phi \right)\left(\phi^{i}\nabla_{i}\phi \right)dA\\ \; & +\mu \int [L^{ij}\nabla_{i}\phi \nabla_{j}\phi -H{\left(\nabla_{s}\phi \right)}^{2}]\psi dA\\ \; & =\int \{\frac{\Delta_{s}G_{H}}{2}+{\bar{\Delta }}_{s}G_{K}+\left(2H^{2}-K\right)G_{H}+2KHG_{K}-2HG\\ \; & +\mu \left[L^{ij}\nabla_{i}\phi \nabla_{j}\phi -H{\left(\nabla_{s}\phi \right)}^{2}\right]\}\psi dA-\int \left[G_{\phi }-\mu \left(\nabla^{2}\phi \right)\right]\left(\phi^{i}\nabla_{i}\phi \right)dA \end{array}$$

In terms of the divergence theorem [46], we have the relation

(13) $$\int dV=\int \frac{\nabla \cdot r}{3}dV=\frac{1}{3}\int n\cdot rdA$$

After a non-trivial lengthy calculation, we obtain the variation of the volume constraint

(14) $$\delta F_{v}=\int P\psi dA$$

### 3.3. Summary of New Helfrich Shape Equations

We have obtained the new shape equations associated with the curvature-component coupling terms in all for the generalized Helfrich free energy. We confirmed that there is no force balance equation in the tangential direction under the framework of general variational principle.

The force balance related to the component variation of membrane surface is

(15) $$G_{\phi }-\mu \Delta_{s}\phi =0$$

and the force balance related to the variation in the normal direction of membrane surface is

(16) $$\begin{array}{cc} \; & \frac{\Delta_{s}G_{H}}{2}+{\bar{\Delta }}_{s}G_{K}+\left(2H^{2}-K\right)G_{H}+2KHG_{K}\\ \; & -2HG+\mu \left(\kappa_{n}-H\right){\left(\nabla_{s}\phi \right)}^{2}+P=0 \end{array}$$

where $\kappa_{n}$ is the normal curvature of a curve passing through any point on the surface and along the direction of $\nabla_{s}\phi$ at that point.

## 4. Special Geometric Shapes with Phase Separation

In this section, we provide several sets of EL equations associated with the composition redistribution on multicomponent membranes for some typical geometric shapes.

### 4.1. Spherical Vesicles

For a spherical membrane with radius *R*, we have $H=\kappa_{n}=-1/R$ and $K=1/R^{2}$. The surface gradient and surface Laplace operators satisfy

(17) $$\begin{array}{cc} \; & \nabla_{s}=\frac{1}{R}\frac{\partial }{\partial \theta }\hat{\theta }+\frac{1}{R\sin\theta }\frac{\partial }{\partial \Psi }\hat{\psi }, \end{array}$$

(18) $$\begin{array}{cc} \; & \Delta_{s}=\frac{1}{R^{2}\sin\theta }\frac{\partial }{\partial \theta }\left(\sin\theta \frac{\partial }{\partial \theta }\right)+\frac{1}{R^{2}\sin^{2}\theta }{\left(\frac{\partial }{\partial \Psi }\right)}^{2}, \end{array}$$

(19) $$\begin{array}{cc} \; & {\bar{\Delta }}_{s}=-\frac{1}{R}\Delta_{s}. \end{array}$$

Therefore, we obtain the relations as follows:

$$\left\{\begin{array}{c} G_{\phi }=\frac{\partial G}{\partial \phi }=\frac{2k^{\prime }\left(\phi \right)}{R^{2}}+\frac{{\bar{k}}^{\prime }\left(\phi \right)}{R^{2}}+\sigma^{\prime }\left(\phi \right)+\lambda \phi \left(1-\phi \right)\left(1-2\phi \right),\\ G_{H}=\frac{\partial G}{\partial H}=-\frac{4k(\phi )}{R},\\ G_{K}=\frac{\partial G}{\partial K}=\bar{k}\left(\phi \right),\\ G=\frac{2k(\phi )}{R^{2}}+\frac{\bar{k}\left(\phi \right)}{R^{2}}+\sigma \left(\phi \right)+\frac{\lambda }{2}\phi^{2}{\left(1-\phi \right)}^{2},\\ \frac{\Delta_{s}G_{H}}{2}=-\frac{2}{R}\Delta_{s}k\left(\phi \right),\\ {\bar{\Delta }}_{s}G_{K}=-\frac{1}{R}\Delta_{s}\bar{k}\left(\phi \right),\\ \left(2H^{2}-K\right)G_{H}=-\frac{4k(\phi )}{R^{3}},\\ 2HKG_{K}=-\frac{2}{R^{3}}\bar{k}\left(\phi \right),\\ -2HG=\frac{2}{R}G=\frac{4k(\phi )}{R^{3}}+\frac{2\bar{k}\left(\phi \right)}{R^{3}}+\frac{2\sigma (\phi )}{R}+\frac{\lambda }{R}\phi^{2}{\left(1-\phi \right)}^{2},\\ \mu \left(\kappa_{n}-H\right){\left(\nabla_{s}\phi \right)}^{2}=0. \end{array}\right.$$

Finally, we obtain the corresponding curvature-component coupling EL equations for spherical membranes as

(20) $$\begin{array}{cc} \; & \frac{1}{R^{2}}\left(2k^{\prime }\left(\phi \right)+{\bar{k}}^{\prime }\left(\phi \right)\right)+\sigma^{\prime }\left(\phi \right)+\lambda \phi \left(1-\phi \right)\left(1-2\phi \right)-\mu \Delta_{s}\phi =0, \end{array}$$

(21) $$\begin{array}{cc} \; & -\frac{1}{R}\Delta_{s}\left[2k\left(\phi \right)+\bar{k}\left(\phi \right)\right]+\frac{2\sigma (\phi )}{R}+\frac{\lambda }{R}\phi^{2}{\left(1-\phi \right)}^{2}+P=0. \end{array}$$

which indicates that for the spherical shape of the EL equation, Equation (21) reveals a key feature of spherical membranes: the equilibrium condition is independent of the compositional gradient ($\nabla_{s}\phi$). This implies that phase separation in spherical vesicles is primarily driven by curvature-modulus coupling rather than spatial heterogeneity.

For binary lipid mixture fluid membranes, in which the bending rigidities of two types of lipids (typically disordered and ordered phases) are very close (the ratio is about 1.25∼2.6 in the experiments [16,49]), we can safely choose $\phi =\phi_{A}$, and the elastic moduli all depend on $\phi_{A}$ defined linearly for simplicity. However, we should note that this linear relation may break down for the binary mixture solid/fluid membranes, such as protein/lipid binary systems [50,51,52,53] and lysolipid/unsaturated lipid binary systems [54,55], for which a relation similar to the constant of Hookean springs in series was proposed [56]. These components actually show the characteristics of a solid or gel/crystalline [57], which can apply the extra shear strain on the membrane and endow the membrane with some distinct properties from the fluid membranes; for example, our previous work [22] found that solid domains spontaneously aggregate on one side of a nearly spherical vesicle and release more area for fluid membranes, and when a micropipette is applied, the fluid membrane is sucked into the pipette, which is probably a reason why the effective bending modulus is soft, as is the fluid component. But in this work, we will focus on only multicomponent fluid membranes. Thus, we have

(22) $$\left\{\begin{array}{c} k\left(\phi_{A}\right)=k_{B}+\left(k_{A}-k_{B}\right)\phi_{A},\\ \bar{k}\left(\phi_{A}\right)={\bar{k}}_{B}+\left({\bar{k}}_{A}-{\bar{k}}_{B}\right)\phi_{A},\\ \sigma \left(\phi_{A}\right)=\sigma_{B}+\left(\sigma_{A}-\sigma_{B}\right)\phi_{A}. \end{array}\right.$$

Then, Equations (20) and (21) can be rewritten as folows:

(23) $$\begin{array}{cc} \; & \frac{1}{R^{2}}\left[2\left(k_{A}-k_{B}\right)+\left({\bar{k}}_{A}-{\bar{k}}_{B}\right)\right]+\left(\sigma_{A}-\sigma_{B}\right)+\lambda \phi_{A}\left(1-\phi_{A}\right)\left(1-2\phi_{A}\right)-\mu \Delta_{s}\phi_{A}=0, \end{array}$$

(24) $$-\frac{1}{R}\left[2\left(k_{A}-k_{B}\right)+\left({\bar{k}}_{A}-{\bar{k}}_{B}\right)\right]\Delta_{s}\phi_{A}+\frac{2}{R}\left[\sigma_{B}+\left(\sigma_{A}-\sigma_{B}\right)\phi_{A}\right]+\frac{\lambda }{R}\phi_{A}^{2}{\left(1-\phi_{A}\right)}^{2}+P=0.$$

and from Equations (23) and (24), we can obtain the equilibrium radius of multicomponent spherical vesicles as

(25) $$\begin{array}{cc} \; & R_{\mathrm{eq}}^{\mathrm{sp}}=\frac{1}{3}(-\frac{K_{2}}{P}+\frac{\sqrt[{3}]{3\sqrt{3}\sqrt{27K_{1}^{2}P^{4}-4K_{1}K_{2}^{3}P^{2}}+27K_{1}P^{2}-2K_{2}^{3}}}{\sqrt[{3}]{2}P}\\ \; & +\frac{\sqrt[{3}]{2}K_{2}^{2}}{P\sqrt[{3}]{3\sqrt{3}\sqrt{27K_{1}^{2}P^{4}-4K_{1}K_{2}^{3}P^{2}}+27K_{1}P^{2}-2K_{2}^{3}}}). \end{array}$$

where $K_{1}=\frac{1}{\mu }{\left[2\left(k_{A}-k_{B}\right)+\left({\bar{k}}_{A}-{\bar{k}}_{B}\right)\right]}^{2}$, and $K_{2}=\frac{2}{R}\left[\sigma_{B}+\left(\sigma_{A}-\sigma_{B}\right)\phi_{A}\right]+\frac{\lambda }{R}\phi_{A}^{2}{\left(1-\phi_{A}\right)}^{2}-\frac{1}{\mu }\left(\sigma_{A}-\sigma_{B}\right)\left[2\left(k_{A}-k_{B}\right)+\left({\bar{k}}_{A}-{\bar{k}}_{B}\right)\right]-\frac{\lambda }{\mu }\phi_{A}\left(1-\phi_{A}\right)\left(1-2\phi_{A}\right)$. When $\phi_{A}=1$ or 0, Equation (25) returns to the classic spherical solution for the homogeneous membrane case [35]. If the transmembrane pressure difference P=0, indicating multicomponent vesicles in an isotonic aqueous environment, then the equilibrium radius is simplified to

(26) $$\begin{array}{c} R_{\mathrm{iso}}^{\mathrm{sp}}=\frac{2\Delta k+\Delta \bar{k}}{\sqrt{2\mu \sigma_{\mathrm{mix}}+\lambda \mu \phi_{A}^{2}{\left(1-\phi_{A}\right)}^{2}-\left[\Delta \sigma +\lambda \phi_{A}\left(1-\phi_{A}\right)\left(1-2\phi_{A}\right)\right]\left[2\Delta k+\Delta \bar{k}\right]}}. \end{array}$$

where $\sigma_{\mathrm{mix}}=\sigma_{B}+\left(\sigma_{A}-\sigma_{B}\right)\phi_{A}$, $\Delta \sigma =\sigma_{A}-\sigma_{B}$, $\Delta k=k_{A}-k_{B}$, and $\Delta \bar{k}={\bar{k}}_{A}-{\bar{k}}_{B}$. $R_{\mathrm{iso}}^{\mathrm{sp}}$ implies that a necessary condition for the existing equilibrium radius of multicomponent vesicles in an isotonic solution is that different membrane components should have different values for the corresponding elastic moduli; otherwise, no multicomponent vesicles can exist in the isotonic aqueous environment. However, the experiments have confirmed that the stable spherical multicomponent vesicles are observed in the isotonic solution [58,59,60]; therefore, on the basis of our model, we can deduce that different lipids should have different bending moduli or different Gaussian moduli.

### 4.2. Cylindrical Vesicles

For a cylindrical membrane with radius *R*, we have H=−1/(2R) and K=0. The surface Laplace operators satisfy

(27) $$\begin{array}{cc} \; & \nabla_{s}=\frac{1}{R}\frac{\partial }{\partial \Psi }\hat{\psi }+\frac{\partial }{\partial z}\hat{z}, \end{array}$$

(28) $$\begin{array}{cc} \; & \Delta_{s}=\frac{1}{R^{2}}{\left(\frac{\partial }{\partial \Psi }\right)}^{2}+{\left(\frac{\partial }{\partial z}\right)}^{2}, \end{array}$$

(29) $$\begin{array}{cc} \; & {\bar{\Delta }}_{s}=0. \end{array}$$

Therefore, we obtain the relations as follows:

$$\left\{\begin{array}{c} G_{\phi }=\frac{\partial G}{\partial \phi }=\frac{k^{\prime }\left(\phi \right)}{2R^{2}}+\sigma^{\prime }\left(\phi \right)+\lambda \phi \left(1-\phi \right)\left(1-2\phi \right),\\ G_{H}=-\frac{2k(\phi )}{R},\\ G_{K}=0,\\ G=\frac{k(\phi )}{2R^{2}}+\sigma \left(\phi \right)+\frac{\lambda }{2}\phi^{2}{\left(1-\phi \right)}^{2},\\ \frac{\Delta_{s}G_{H}}{2}=-\frac{1}{R}\Delta_{s}k\left(\phi \right),\\ {\bar{\Delta }}_{s}G_{K}=0,\\ \left(2H^{2}-K\right)G_{H}=-\frac{k(\phi )}{R^{3}},\\ -2HG=\frac{k(\phi )}{2R^{3}}+\frac{\sigma (\phi )}{R}+\frac{\lambda }{2R}\phi^{2}{\left(1-\phi \right)}^{2},\\ \mu \left(\kappa_{n}-H\right){\left(\nabla_{s}\phi \right)}^{2}=\mu \left(\kappa_{n}+\frac{1}{2R}\right){\left(\nabla_{s}\phi \right)}^{2}. \end{array}\right.$$

Finally, we obtain the corresponding curvature-component coupling EL equations for cylindrical membranes as

(30) $$\begin{array}{cc} \; & \frac{k^{\prime }\left(\phi \right)}{2R^{2}}+\sigma^{\prime }\left(\phi \right)+\lambda \phi \left(1-\phi \right)\left(1-2\phi \right)-\mu \Delta_{s}\phi =0, \end{array}$$

(31) $$\begin{array}{cc} \; & -\frac{1}{R}\Delta_{s}k\left(\phi \right)-\frac{k(\phi )}{2R^{3}}+\frac{\sigma (\phi )}{R}+\frac{\lambda }{2R}\phi^{2}{\left(1-\phi \right)}^{2}+\mu \left(\kappa_{n}+\frac{1}{2R}\right){\left(\nabla_{s}\phi \right)}^{2}+P=0. \end{array}$$

Following the definitions of the elastic moduli for the spherical shape, Equations (30) and (31) can be rewritten as folows:

(32) $$\begin{array}{cc} \; & \frac{1}{R^{2}}\left(k_{A}-k_{B}\right)+\left(\sigma_{A}-\sigma_{B}\right)+\lambda \phi_{A}\left(1-\phi_{A}\right)\left(1-2\phi_{A}\right)-\mu \Delta_{s}\phi_{A}=0,\\ \; & -\frac{2}{R}\left(k_{A}-k_{B}\right)\Delta_{s}\phi_{A}-\frac{1}{2R^{3}}\left[k_{B}+\left(k_{A}-k_{B}\right)\phi_{A}\right]+\frac{1}{R}\left[\sigma_{B}+\left(\sigma_{A}-\sigma_{B}\right)\phi_{A}\right] \end{array}$$

(33) $$\begin{array}{cc} \; & +\frac{\lambda }{2R}\phi_{A}^{2}{\left(1-\phi_{A}\right)}^{2}+\mu \left(\kappa_{n}+\frac{1}{2R}\right){\left(\nabla_{s}\phi \right)}^{2}+P=0. \end{array}$$

and from Equations (32) and (33), we can obtain the equation for the equilibrium radius of multicomponent cylindrical vesicles as

(34) $$\begin{array}{cc} \; & -\frac{2}{\mu R^{3}}{\left(k_{A}-k_{B}\right)}^{2}-\frac{2}{\mu R}\left(k_{A}-k_{B}\right)\left(\sigma_{A}-\sigma_{B}\right)-\frac{2\lambda }{\mu R}\left(k_{A}-k_{B}\right)\phi_{A}\left(1-\phi_{A}\right)\left(1-2\phi_{A}\right)\\ \; & -\frac{1}{2R^{3}}\left[k_{B}+\left(k_{A}-k_{B}\right)\phi_{A}\right]+\frac{1}{R}\left[\sigma_{B}+\left(\sigma_{A}-\sigma_{B}\right)\phi_{A}\right]\\ \; & +\frac{\lambda }{2R}\phi_{A}^{2}{\left(1-\phi_{A}\right)}^{2}+\mu \left(\kappa_{n}+\frac{1}{2R}\right){\left(\nabla_{s}\phi \right)}^{2}+P=0. \end{array}$$

If the transmembrane pressure difference P=0, indicating multicomponent vesicles in an isotonic aqueous environment, and if the component Lipid A is uniformly distributed on the cylindrical surface, then the equilibrium radius is simplified to

(35) $$\begin{array}{c} R_{\mathrm{iso}}^{\mathrm{cy}}=\sqrt{\frac{\mu k_{\mathrm{mix}}+4{\left(\Delta k\right)}^{2}}{2\mu \sigma_{\mathrm{mix}}+\lambda \mu \phi_{A}^{2}{\left(1-\phi_{A}\right)}^{2}-4\Delta k\left[\Delta \sigma +\lambda \phi_{A}\left(1-\phi_{A}\right)\left(1-2\phi_{A}\right)\right]}}. \end{array}$$

where $k_{\mathrm{mix}}=k_{B}+\left(k_{A}-k_{B}\right)\phi_{A}$, and $\Delta \sigma =\sigma_{A}-\sigma_{B}$.

### 4.3. Vesicles with Constant Mean Curvature

For a membrane with the constant mean curvature $H_{0}$, we have $H=H_{0}$. Therefore, we obtain the relations as follows:

$$\left\{\begin{array}{c} G_{\phi }=2k^{\prime }\left(\phi \right)H_{0}^{2}+{\bar{k}}^{\prime }\left(\phi \right)K+\sigma^{\prime }\left(\phi \right)+\lambda \phi \left(1-\phi \right)\left(1-2\phi \right),\\ G_{H}=4k\left(\phi \right)H_{0},\\ G_{K}=\frac{\partial G}{\partial K}=\bar{k}\left(\phi \right),\\ G=2k\left(\phi \right)H_{0}^{2}+\bar{k}\left(\phi \right)K+\sigma \left(\phi \right)+\frac{\lambda }{2}\phi^{2}{\left(1-\phi \right)}^{2},\\ \frac{\Delta_{s}G_{H}}{2}=2H_{0}\Delta_{s}k\left(\phi \right),\\ {\bar{\Delta }}_{s}G_{K}={\bar{\Delta }}_{s}\bar{k}\left(\phi \right),\\ \left(2H^{2}-K\right)G_{H}=4k\left(\phi \right)H_{0}\left(2H_{0}^{2}-K\right),\\ 2HKG_{K}=2KH_{0}\bar{k}\left(\phi \right),\\ -2HG=-2H_{0}G=-4k\left(\phi \right)H_{0}^{3}-2\bar{k}\left(\phi \right)KH_{0}-2\sigma \left(\phi \right)H_{0}-\lambda H_{0}\phi^{2}{\left(1-\phi \right)}^{2},\\ \mu \left(\kappa_{n}-H\right){\left(\nabla_{s}\phi \right)}^{2}=\mu \left(\kappa_{n}-H_{0}\right){\left(\nabla_{s}\phi \right)}^{2}. \end{array}\right.$$

Finally, we obtain the corresponding curvature-component coupling EL equations for spherical membranes as

(36) $$\begin{array}{cc} \; & 2k^{\prime }\left(\phi \right)H_{0}^{2}+{\bar{k}}^{\prime }\left(\phi \right)K+\sigma^{\prime }\left(\phi \right)+\lambda \phi \left(1-\phi \right)\left(1-2\phi \right)-\mu \Delta_{s}\phi =0, \end{array}$$

(37) $$\begin{array}{cc} \; & 2H_{0}\Delta_{s}k\left(\phi \right)+{\bar{\Delta }}_{s}\bar{k}\left(\phi \right)-4k\left(\phi \right)KH_{0}-2\sigma \left(\phi \right)H_{0}-\lambda H_{0}\phi^{2}{\left(1-\phi \right)}^{2}\\ \; & +\mu \left(\kappa_{n}-H_{0}\right){\left(\nabla_{s}\phi \right)}^{2}+P=0 \end{array}$$

If $H_{0}=0$, which is usually called a minimal surface in differential geometry, then the equations above can be reduced to

(38) $$\begin{array}{cc} \; & {\bar{k}}^{\prime }\left(\phi \right)K+\sigma^{\prime }\left(\phi \right)+\lambda \phi \left(1-\phi \right)\left(1-2\phi \right)-\mu \Delta_{s}\phi =0, \end{array}$$

(39) $$\begin{array}{cc} \; & {\bar{\Delta }}_{s}\bar{k}\left(\phi \right)+\mu \kappa_{n}{\left(\nabla_{s}\phi \right)}^{2}+P=0 \end{array}$$

which define a set of new EL equations to describe the multicomponent minimal surface or the minimal surface with diffusing components. This enriches the research objects in the field of differential geometry and geometric analysis.

## 5. Discussion and Perspectives

In the previous work done by Jülicher and Lipowsky [39], the extra term, the line tension, was introduced to describe two-component membranes with a strong segregation limit, where the components are not mixed, and there is a well-defined boundary between two domains. This requests the corresponding matching conditions related to the boundary between the domains, and later, these matching conditions were naturally derived by Yang et al. [61]. Our work allows us to study physical phenomena associated with phase separation in multicomponent membranes, spanning from weak segregation to strong segregation, where the latter represents a limiting case. For example, how does the competition between the redistribution of the lipid composition and the curvature-driven lipid sorting determine the formation and growth of the disordered lipid domains? The equilibrium states of membrane shape and lipid composition distribution with the global spherical topology can be investigated, since the pressure energy term is appropriately included. The pressure term, neglected in many previous studies [29,30,31,32,33,34], is an important part to calculate the energy barrier of tubular formation [62]. Without this term, only the local membrane patch can be modeled. The significance of two geometric constraints—the surface tension term and the pressure term—has been explained in Section 2.2. They must be considered together to obtain the correct estimations for changes in global vesicle morphology. For instance, a large amount of cell systems, such as membrane protrusions [63,64], adhesion points [63,65], and bacterial membranes [66], contain functional microdomains on the membrane exhibiting controllable deformation (curvature change) associated with lipid redistribution. In our case, the transition from a domain rich in one component to the domain rich in another component is smooth, and therefore, adding the line energy is not necessary. The current approach can naturally describe the formation of fission/fusion regions without assuming the existence of two membrane domains with two lipid compositions A and B in advance and imposing the narrowing of the membrane neck.

We can further modify the current model to study more aspects of membrane phenomena, such as a direct extension on the budding of multicomponent vesicles, extending our recent work on single-component vesicles [67]. We plan to introduce the adhesion energy term $F_{\mathrm{adh}}=\int \gamma \left(\phi \right)dA$ in subsequent work to study the effect of membrane–substrate or membrane–membrane adhesion on phase separation [68], and the specific implementation will be parameterized in combination with experimental data (such as AFM force curves). Our framework provides a foundation for modeling realistic multicomponent cells [69,70,71,72], particularly in scenarios involving large deformations (e.g., amoeboid motion or phagocytosis). Electrostatic interactions between different charged lipid components will be another interesting effect that can be combined with the current model to complete the free energy of multicomponent membranes, in which electrostatic elastic coupling could play an important role [73,74,75]. In addition to the known membrane fusion process between oppositely charged phospholipid bilayers [76], our new theory could predict that a two-dimensional colloidal lattice formed by like-charged lipid or protein clusters could trigger another type of like-charged lipid in the lattice gap to aggregate due to elastically induced attractive interactions on microdomain-separated membranes, thereby effectively reducing the surface tension to facilitate the vesicle shape changes and the occurrence of membrane fusion. The magnetic field is gradually realized as an increasingly key factor in affecting changes in membrane shape [77]; therefore, we hope to include the external magnetic field in our model in the following work. Mathematically, we should be concerned with membranes with an or more open edges [78,79], enriching the geometric objects with novel interactions from the edges. While our model provides a comprehensive framework for multicomponent membranes, several limitations should be noted. First, the assumption of linear dependence of elastic moduli on composition may oversimplify the nonlinear coupling effects observed in real systems. Second, dynamic processes such as lipid flip-flop or protein diffusion are not included in the current equilibrium formulation.

Mathematically, inspired by the original Helfrich functional Nitsche has investigated, the new periodic extremal surface problems have emphasized its importance [80]. Therefore, the development of such a continuous energy functional is not only a pressing need in physics and biology, but also enriches and extends the mathematical theory on the gradient flow for Willmore functional [81,82] to the generalized Helfrich functional (Helfrich flow). Some future challenges have been proposed and concluded in [83,84].

In the current work, we present a generalized Helfrich free-energy framework for multicomponent fluid membranes that unifies curvature elasticity, composition-dependent phase separation, and geometric constraints. By incorporating spatially varying bending rigidity k(ϕ), Gaussian modulus $\bar{k}\left(\phi \right)$, and membrane tension σ(ϕ), we derive for the first time a set of curvature-component coupled Euler–Lagrange equations governing both membrane shape evolution and lateral composition redistribution. Crucially, our model resolves the limitations of prior approaches by explicitly accounting for the competition between area and volume constraints, which generates multiple equilibrium morphologies (e.g., spheres, cylinders, etc.) as local free-energy minima. Analytical solutions for isotonic vesicles reveal that membrane stability requires distinct elastic moduli among components ($k_{A}\neq k_{B}$, ${\bar{k}}_{A}\neq {\bar{k}}_{B}$), consistent with experimental observations of coexisting lipid domains. The Ginzburg–Landau double-well potential $V\left(\phi \right)=\left(\lambda /2\right)\phi^{2}{\left(1-\phi \right)}^{2}$ quantitatively links the coupling coefficient λ to interfacial tension between phases. The parameter λ is central to linking thermodynamic phase behavior and membrane mechanics. Its value dictates phase separation strength, interfacial tension, and dynamic responses. Our incorporation of λ via the double-well potential provides a universal framework for studying multicomponent membrane morphology, with generalized mixing rules proposed for N components in broader applications. The new Laplace–Beltrami operator ${\bar{\Delta }}_{s}$ related to local topology in our model demonstrates new predictive capabilities distinct from the existing models. Our new theoretical framework establishes a foundation and baseline for probing membrane remodeling mechanisms in cellular processes and synthetic vesicles, with direct extensions to active systems under external fields.

## Data Availability

The data that support the findings of this study are available from the corresponding author upon reasonable request.

## References

[1] Seifert U. (1997). Configurations of fluid membranes and vesicles. Adv. Phys..

[2] Dimova R., Marques C. (2019). The Giant Vesicle Book.

[3] Simons K., Ikonen E. (1997). Functional rafts in cell membranes. Nature.

[4] Rajendran L., Simons K. (2005). Lipid rafts and membrane dynamics. J. Cell Sci..

[5] Jacobson K., Dietrich C. (1999). Looking at lipid rafts?. Trends Cell Biol..

[6] Wu H., Noguchi H. (2013). Effects of anchored flexible polymers on mechanical properties of model biomembranes. AIP Conference Proceedings.

[7] Wu H., Shiba H., Noguchi H. (2013). Mechanical properties and microdomain separation of fluid membranes with anchored polymers. Soft Matter.

[8] McIntosh T.J., Vidal A., Simon S.A. (2003). Sorting of lipids and transmembrane peptides between detergent-soluble bilayers and detergent-resistant rafts. Biophys. J..

[9] Needham D., Nunn R.S. (1990). Elastic deformation and failure of lipid bilayer membranes containing cholesterol. Biophys. J..

[10] Grzybek M., Kozubek A., Dubielecka P., Sikorski A.F. (2005). Rafts-the current picture. Folia Histochem. Cytobiol..

[11] Allende D., Vidal A., McIntosh T.J. (2004). Jumping to rafts: Gatekeeper role of bilayer elasticity. Trends Biochem. Sci..

[12] Dietrich C., Bagatolli L., Volovyk Z., Thompson N., Levi M., Jacobson K., Gratton E. (2001). Lipid rafts reconstituted in model membranes. Biophys. J..

[13] Simons K., Van Meer G. (1988). Lipid sorting in epithelial cells. Biochemistry.

[14] Thompson T., Tillack T.W. (1985). Organization of glycosphingolipids in bilayers and plasma membranes of mammalian cells. Annu. Rev. Biophys. Biophys. Chem..

[15] Brown R.E. (1998). Sphingolipid organization in biomembranes: What physical studies of model membranes reveal. J. Cell Sci..

[16] Baumgart T., Hess S.T., Webb W.W. (2003). Imaging coexisting fluid domains in biomembrane models coupling curvature and line tension. Nature.

[17] Veatch S.L., Keller S.L. (2002). Organization in lipid membranes containing cholesterol. Phys. Rev. Lett..

[18] Käs J., Sackmann E. (1991). Shape transitions and shape stability of giant phospholipid vesicles in pure water induced by area-to-volume changes. Biophys. J..

[19] Döbereiner H., Käs J., Noppl D., Sprenger I., Sackmann E. (1993). Budding and fission of vesicles. Biophys. J..

[20] Edidin M. (1997). Lipid microdomains in cell surface membranes. Curr. Opin. Struct. Biol..

[21] Jacobson K., Sheets E.D., Simson R. (1995). Revisiting the fluid mosaic model of membranes. Science.

[22] Xin W., Wu H., Grason G.M., Santore M.M. (2021). Switchable positioning of plate-like inclusions in lipid membranes: Elastically mediated interactions of planar colloids in 2D fluids. Sci. Adv..

[23] Parton R.G. (1996). Caveolae and caveolins. Curr. Opin. Cell Biol..

[24] Li C., Perez Y.Q., Lamaze C., Blouin C.M. (2024). Lipid nanodomains and receptor signaling: From actin-based organization to membrane mechanics. Curr. Opin. Cell Biol..

[25] Helfrich W. (1973). Elastic properties of lipid bilayers: Theory and possible experiments. Z. für Naturforschung C.

[26] Iglič A. (1997). A possible mechanism determining the stability of spiculated red blood cells. J. Biomech..

[27] Wu H., De León M.A.P., Othmer H.G. (2018). Getting in shape and swimming: The role of cortical forces and membrane heterogeneity in eukaryotic cells. J. Math. Biol..

[28] Illya G., Lipowsky R., Shillcock J. (2006). Two-component membrane material properties and domain formation from dissipative particle dynamics. J. Chem. Phys..

[29] Taniguchi T. (1996). Shape deformation and phase separation dynamics of two-component vesicles. Phys. Rev. Lett..

[30] Jiang Y., Lookman T., Saxena A. (2000). Phase separation and shape deformation of two-phase membranes. Phys. Rev. E.

[31] Jiang H., Powers T.R. (2008). Curvature-driven lipid sorting in a membrane tubule. Phys. Rev. Lett..

[32] Rautu S.A., Rowlands G., Turner M.S. (2015). Membrane composition variation and underdamped mechanics near transmembrane proteins and coats. Phys. Rev. Lett..

[33] Dharmavaram S., She S.B., Lázaro G., Hagan M.F., Bruinsma R. (2019). Gaussian curvature and the budding kinetics of enveloped viruses. PLoS Comput. Biol..

[34] Janssen M., Liese S., Al-Izzi S.C., Carlson A. (2024). Stability of a biomembrane tube covered with proteins. Phys. Rev. E.

[35] Ou-Yang Z.C., Helfrich W. (1989). Bending energy of vesicle membranes: General expressions for the first, second, and third variation of the shape energy and applications to spheres and cylinders. Phys. Rev. A.

[36] Bray A.J. (2002). Theory of phase-ordering kinetics. Adv. Phys..

[37] Takiue T. (2022). Heterogeneity and deformation behavior of lipid vesicles. Curr. Opin. Colloid Interface Sci..

[38] De Gennes P.G. (1979). Scaling Concepts in Polymer Physics.

[39] Jülicher F., Lipowsky R. (1996). Shape transformations of vesicles with intramembrane domains. Phys. Rev. E.

[40] Qin R., Bhadeshia H. (2010). Phase field method. Mater. Sci. Technol..

[41] Simons K., Ehehalt R. (2002). Cholesterol, lipid rafts, and disease. J. Clin. Investig..

[42] Capovilla R., Guven J. (2002). Stresses in lipid membranes. J. Phys. A Math. Gen..

[43] Miao L., Lomholt M.A., Kleis J. (2002). Dynamics of shape fluctuations of quasi-spherical vesicles revisited. Eur. Phys. J. E.

[44] Müller M.M., Deserno M., Guven J. (2005). Geometry of surface-mediated interactions. EPL (Europhys. Lett.).

[45] Capovilla R., Guven J., Santiago J. (2003). Deformations of the geometry of lipid vesicles. J. Phys. A Math. Gen..

[46] Tu Z., Ou-Yang Z.C. (2004). A geometric theory on the elasticity of bio-membranes. J. Phys. A Math. Gen..

[47] Wu H., Tu Z. (2009). Theoretical and numerical investigations on shapes of planar lipid monolayer domains. J. Chem. Phys..

[48] Villanueva M.E., Bar L., Redondo-Morata L., Namdar P., Ruysschaert J.M., Pabst G., Vandier C., Bouchet A.M., Losada-Pérez P. (2024). Spontaneous nanotube formation of an asymmetric glycolipid. J. Colloid Interface Sci..

[49] Roux A., Cuvelier D., Nassoy P., Prost J., Bassereau P., Goud B. (2005). Role of curvature and phase transition in lipid sorting and fission of membrane tubules. EMBO J..

[50] Bouvrais H., Méléard P., Pott T., Jensen K.J., Brask J., Ipsen J.H. (2008). Softening of POPC membranes by magainin. Biophys. Chem..

[51] Capraro B.R., Yoon Y., Cho W., Baumgart T. (2010). Curvature sensing by the epsin N-terminal homology domain measured on cylindrical lipid membrane tethers. J. Am. Chem. Soc..

[52] Otten D., Brown M.F., Beyer K. (2000). Softening of membrane bilayers by detergents elucidated by deuterium NMR spectroscopy. J. Phys. Chem. B.

[53] Pan J., Tieleman D.P., Nagle J.F., Kučerka N., Tristram-Nagle S. (2009). Alamethicin in lipid bilayers: Combined use of X-ray scattering and MD simulations. Biochim. Biophys. Acta (BBA)-Biomembr..

[54] Henriksen J.R., Andresen T.L., Feldborg L.N., Duelund L., Ipsen J.H. (2010). Understanding detergent effects on lipid membranes: A model study of lysolipids. Biophys. J..

[55] Melero A., Chiaruttini N., Karashima T., Riezman I., Funato K., Barlowe C., Riezman H., Roux A. (2018). Lysophospholipids facilitate COPII vesicle formation. Curr. Biol..

[56] Bashkirov P.V., Kuzmin P.I., Vera Lillo J., Frolov V.A. (2022). Molecular shape solution for mesoscopic remodeling of cellular membranes. Annu. Rev. Biophys..

[57] Chen D., Santore M.M. (2014). Large effect of membrane tension on the fluid–solid phase transitions of two-component phosphatidylcholine vesicles. Proc. Natl. Acad. Sci. USA.

[58] Russell J.T. (1981). The isolation of purified neurosecretory vesicles from bovine neurohypophysis using isoosmolar density gradients. Anal. Biochem..

[59] Hallett F.R., Marsh J., Nickel B.G., Wood J.M. (1993). Mechanical properties of vesicles. II. A model for osmotic swelling and lysis. Biophys. J..

[60] Yang P., Lipowsky R., Dimova R. (2009). Nanoparticle formation in giant vesicles: Synthesis in biomimetic compartments. Small.

[61] Yang P., Du Q., Tu Z. (2017). General neck condition for the limit shape of budding vesicles. Phys. Rev. E.

[62] Li Y., Lipowsky R., Dimova R. (2011). Membrane nanotubes induced by aqueous phase separation and stabilized by spontaneous curvature. Proc. Natl. Acad. Sci. USA.

[63] Gaus K., Gratton E., Kable E.P., Jones A.S., Gelissen I., Kritharides L., Jessup W. (2003). Visualizing lipid structure and raft domains in living cells with two-photon microscopy. Proc. Natl. Acad. Sci. USA.

[64] Heijnen H., Van Lier M., Waaijenborg S., Ohno-Iwashita Y., Waheed A., Inomata M., Gorter G., Möbius W., Akkerman J., Slot J. (2003). Concentration of rafts in platelet filopodia correlates with recruitment of c-Src and CD63 to these domains. J. Thromb. Haemost..

[65] Gaus K., Le Lay S., Balasubramanian N., Schwartz M.A. (2006). Integrin-mediated adhesion regulates membrane order. J. Cell Biol..

[66] Epand R.M., Epand R.F. (2009). Lipid domains in bacterial membranes and the action of antimicrobial agents. Biochim. Biophys. Acta (BBA)-Biomembr..

[67] Wu H., Ou-Yang Z.C., Podgornik R. (2023). A Note on Vestigial Osmotic Pressure. Membranes.

[68] Bibissidis N., Betlem K., Cordoyiannis G., Prista-von Bonhorst F., Goole J., Raval J., Daniel M., Góźdź W., Iglič A., Losada-Pérez P. (2020). Correlation between adhesion strength and phase behaviour in solid-supported lipid membranes. J. Mol. Liq..

[69] Wu H., Thiébaud M., Hu W.F., Farutin A., Rafai S., Lai M.C., Peyla P., Misbah C. (2015). Amoeboid motion in confined geometry. Phys. Rev. E.

[70] Wu H., Farutin A., Hu W.F., Thiébaud M., Rafaï S., Peyla P., Lai M.C., Misbah C. (2016). Amoeboid swimming in a channel. Soft Matter.

[71] Wang Q., Wu H. (2021). Mathematical modeling of chemotaxis guided amoeboid cell swimming. Phys. Biol..

[72] Farutin A., Wu H., Hu W.F., Rafaï S., Peyla P., Lai M.C., Misbah C. (2019). Analytical study for swimmers in a channel. J. Fluid Mech..

[73] Wu H., Ou-Yang Z.C., Podgornik R. (2024). Electrostatic-elastic coupling in colloidal crystals. Europhys. Lett..

[74] Wu H., Ou-Yang Z.C., Podgornik R. (2025). Continuum theory of electrostatic-elastic coupling interactions in colloidal crystals. Commun. Theor. Phys..

[75] Liu J., Wu Y., Li Y., Yang L., Wu H., He Q. (2023). Rotary biomolecular motor-powered supramolecular colloidal motor. Sci. Adv..

[76] Pantazatos D., MacDonald R. (1999). Directly observed membrane fusion between oppositely charged phospholipid bilayers. J. Membr. Biol..

[77] Deng Y.X., Liu Y., Shu Y.G., Ou-Yang Z.C. (2018). Deformation theory of polymersome magneto-valves. Europhys. Lett..

[78] Capovilla R., Guven J., Santiago J. (2002). Lipid membranes with an edge. Phys. Rev. E.

[79] Tu Z., Ou-Yang Z. (2003). Lipid membranes with free edges. Phys. Rev. E.

[80] Nitsche J.C. (1993). Periodic surfaces that are extremal for energy functionals containing curvature functions. Statistical Thermodynamics and Differential Geometry of Microstructured Materials.

[81] Kuwert E., Schätzle R. (2002). Gradient flow for the Willmore functional. Commun. Anal. Geom..

[82] Kuwert E., Schätzle R. (2004). Removability of point singularities of Willmore surfaces. Ann. Math..

[83] Tu Z. (2013). Challenges in the theoretical investigations of lipid membrane configurations. Chin. Phys. B.

[84] Tu Z., Ou-Yang Z. (2014). Recent theoretical advances in elasticity of membranes following Helfrich’s spontaneous curvature model. Adv. Colloid Interface Sci..

